# Editorial: Genetics of Acquired Antimicrobial Resistance in Animal and Zoonotic Pathogens

**DOI:** 10.3389/fmicb.2017.02428

**Published:** 2017-12-05

**Authors:** Axel Cloeckaert, Michel S. Zygmunt, Benoît Doublet

**Affiliations:** ISP, Institut National de la Recherche Agronomique, Université François Rabelais de Tours, UMR 1282, Nouzilly, France

**Keywords:** one health, integron, mobile genetic element, antibiotic resistance, dissemination

Antimicrobial resistance has become a global public health concern due to multidrug-resistant (MDR) bacteria and to the lack of novel antibiotics. Resistant bacteria, including zoonotic pathogens, can be exchanged between animals and humans through direct contact, the food chain, or contamination of the shared environment. Resistance to medically-important antibiotics such as extended spectrum beta-lactams, carbapenems, fluoroquinolones, or aminoglycosides, is of increasing magnitude among zoonotic pathogens. Acquired antimicrobial resistance is the result of an evolutionary process by which microorganisms adapt to antibiotics through several mechanisms including alteration of drug target by mutations and horizontal transfer of novel/foreign genes, referred to as resistance genes. Acquired resistance genes coding for any of the three major resistance mechanisms, i.e., enzymatic inactivation, reduced intracellular accumulation, or modification of the cellular target sites are associated with mobile genetic elements including plasmids, transposons, gene cassettes, integrative, and conjugative elements or other mobile elements(Schwarz et al., 2017).

This Research Topic is focused on acquired antimicrobial resistance mechanisms in animal and zoonotic pathogens isolated from food-producing animals, food products, companion animals, humans, and the environment. Different sets of articles document the most important aspects of the genetics of acquired antimicrobial resistance extending from medically-important resistance genes such as those conferring resistance to extended spectrum cephalosporins and more recently those conferring resistance to colistin, emerging epidemic clones, primary mobile genetic elements such as class 1 integrons, culture-independent approach of resistomes, dissemination between animals and humans, to the “One Health” concept.

First, in an elegant review article, Imperial and Ibana addressed the global problem of the spread of emerging antibiotic-resistant bacteria in the “One Health” perspective. They summarized the processes that govern the spread of antibiotic resistance in relation to resistance genes, their horizontal transfer through mobile genetic elements, the microbial ecology of resistant bacteria in the human gut microbiota, and their dissemination by international travel of humans, animals, or food. Finally, they discussed the probiotic use in both human and veterinary applications to tackle the antibiotic resistance threat and concluded on the “double-edged sword” with potential risk in propagating antibiotic resistance by probiotics.

*Salmonella enterica* spp. are important zoonotic pathogens related to foodborne diseases worldwide. In the present Research Topic, Zhao et al. and Zhao et al. contributed with two research articles dealing with antimicrobial resistance of *S. enterica* serotypes isolated from food-producing animals (chickens, ducks, and pigs) in farms and slaughterhouses in the Shandong province, China. They reported a higher prevalence of *Salmonella* in chickens (~24%) compared to pigs (~9%). The serotype distribution and multidrug resistance phenotypes were dependent on the animal species, serotypes Indiana, and Enteritidis being prevalent in chickens and strongly associated with MDR phenotypes. High antimicrobial resistance rates were observed for old class antibiotics such as nalidixic acid, ampicillin, and tetracyclines (>80%). The plasmid-mediated quinolone resistance genes *qnr* were also frequently detected in isolates from farm animals and in slaughterhouses contributing probably with target gene mutations to a rate of around 40% of ciprofloxacin resistance. A relatively high resistance rate to cefotaxime (~30%) was also described, mostly *Salmonella* isolates harboring the ESBL gene *bla*_CTX-M-55_, largely predominant in Asia. In another study from China, Huang et al. investigated the prevalence of colistin resistance and associated *mcr-1* gene on a large collection of commensal *E. coli* isolates (>4,000) from food-producing animals during the period 2013–2014. They reported an overall resistance rate of 18.7% with MIC colistin ≥ 4 mg/L, with a higher frequency of colistin-resistant *E. coli* isolated from pigs (24%) compared to chickens (14%). Among 200 randomly selected colistin-resistant *E. coli* isolates, they found 182 positive *mcr-1* positive isolates. Colistin being in some cases the last therapeutic option to treat infections due to carbapenemase-producing Gram-negative bacteria, the high prevalence of MCR-1-mediated colistin resistance among commensal *E. coli* recovered from food-producing animals is worrisome in China. These MCR-1-producing *Enterobacteriaceae* might transfer to humans through the food chain or to farmers via direct animal contact, and furthermore transfer the *mcr-1* resistance gene to human pathogens. Since the recent ban of colistin as animal feed additive in China, future surveillance programmes will be necessary to follow the evolution of colistin resistance in food-producing animals(Walsh and Wu, 2016).

This Research Topic includes also a panel of articles focused on resistance genes in different companion animal pathogens. Resistance to extended spectrum cephalosporins is a major concern for companion animals due to the close contact with their owners. Liu et al. investigated the occurrence of Extended Spectrum Beta Lactamase (ESBL)-producing clinical *E. coli* recovered from dogs and cats in the United States, from 2009 to 2013. They reported a prevalence of 3.8% ESBL-producing *E. coli*, the *bla*_CTX-M_ resistance genes being the most prevalent ones followed by the cephalosporinase and carbapenemase genes, *bla*_CMY-2_ and *bla*_OXA-48_, respectively. CTX-M-producing MDR *E. coli* isolates were primarily of sequence types ST131, ST648, and ST405 suggesting the possible transfer of predominant human clones with companion animals in the U.S. In a similar large epidemiological study dealing with *Morganellaceae* of animal origin in France, Schultz et al. investigated the prevalence of ESBL and carbapenemase genes and their genetic supports. They found a similar prevalence of ESBL producers (~4%) among *Proteus mirabilis* isolates from dogs, cats, and horses. Interestingly, molecular characterization showed the spread of a clonal population harboring the MDR integrative mobilizable element SGI1-V (*Salmonella* genomic island 1—variant V) carrying the ESBL gene *bla*_VEB-6_, previously identified in human isolates in France. Xia et al. reported a study focused on 16S rRNA methylases conferring high-level resistance to aminoglycosides in *Klebsiella pneumoniae* isolates from diseased dogs and cats in a veterinary hospital in China. They highlighted the spread of genetically-related *K. pneumoniae* ST37 isolates as well as the horizontal transfer of a conjugative IncF33:A-:B- plasmid carrying the 16S rRNA methylase gene *rmtB* and the ESBL gene *bla*_CTX-M-55_. Finally, Deng et al. described the multiresistance genes *vga*(A), *vga*(A)_LC_, *sal*(A), and *lsa*(E) carried by plasmids and chromosomal genomic islands and conferring resistance to pleuromutilins, lincosamides, and streptogramin A in different *Staphylococci* species isolated from pet animals and sometimes their owners in China. In regard of all these articles, it is important to consider the current role of companion animals as a reservoir of resistant bacteria and their mobile resistance determinants that may be exchanged in either direction between animals and humans, warranting the prudent use of all antibiotics in veterinary medicine of companion animals.

Acquired resistance mechanisms are based on resistance mutations of a chromosomal gene or on the acquisition of mobile resistance genes. Several articles exemplified both phenomena of molecular resistance mechanisms, whose relative importance depends on the antibiotic family and the implicated bacterial species. With the recent description of plasmid-mediated resistance to colistin (*mcr* genes), a renewed interest of research took place for molecular resistance mechanisms to polymixins. Prasgasam et al. characterized carbapenem- and colistin-resistant *K. pneumoniae* isolates from bacteremia cases in India by whole genome sequencing. They described multiple mutations in the chromosomal genes coding for lipopolysaccharide (LPS) lipid A modifications including silent mutations, point mutations, insertions and/or deletions. The most significant were mutations in the *mgrB* gene. The resistance genes *mcr* were not detected in this study. The significance of other mutations observed in this study needs to be confirmed for conferring polymixin resistance. Another example of resistance-conferring mutations in chromosomal genes is given in the study by Xu et al. dealing with fosfomycin resistance in *Staphylococcus aureus*. The key resistance mechanisms to fosfomycin include the production of fosfomycin-modifying enzymes (FosA, FosB, FosC, and FosX), modifications of the target enzyme MurA or the membrane transporters GlpT and UhpT. The GlpT and UhpT transporters are responsible for fosfomycin uptake. They previously showed that mutations in *glpT* and *uhpT* genes were common in fosfomycin- and methicillin-resistant *S. aureus* (Fu et al., 2016). Using *uhpT* and/or *glpT* deletion mutants in *S. aureus*, they confirmed the role of mutations or insertional inactivation in these transporter or their regulatory genes in high-level fosfomycin resistance (MIC > 1,024 μg/ml). Cha et al. investigated the phylogenetic lineages of fluoroquinolone- and macrolide-resistant *Campylobacter jejuni* isolates recovered from Michigan patients. Resistance mechnisms to fluoroquinolones and macrolides involve acquisition of mutations of the target sites of the antibiotics, i.e., the DNA gyrase/topoisomerase IV and 23S rRNA subunit, respectively. They identified clonal spread of specific fluoroquinolone-resistant *C. jejuni* lineages, like ST464, associated with history of foreign travel and also a significant association between tetracycline-resistant *C. jejuni* ST982 and contact with cattle, chickens and drinking well water at home. Linking genetic diversity and antimicrobial resistance profiles of *C. jejuni* from various sources is needed to better understand transmission dynamics to humans.

Acquired resistance genes are not by themselves mobile but are carried by various genetic structures allowing their horizontal mobility at the molecular or cellular level. The spread of successful multidrug resistance (MDR) mobile genetic elements (plasmids and genomic islands) between bacteria is the main driving force in the dissemination of acquired antibiotic resistance genes. The involvement of plasmids in the zoonotic spread of ESBL- and carbapenemase-resistance genes between human and animal reservoirs are documented by several articles in the present Research Topic. Seiffert et al. described the occurrence of a new plasmid variant IncK2 carrying the cephalosporinase gene *bla*_CMY-2_ in *E. coli* isolated from poultry, poultry meat and humans. The identification of very close CMY-2-encoding IncK2 plasmids in genetically diverse *E. coli* from poultry and humans suggested an important ability of horizontal transfer by conjugation in different reservoirs. In another article also dealing with ESBL-producing *Enterobacteriaceae*, Moremi et al. analysed ESBL producers in wild fish obtained from Lake Victoria as well as in environmental samples obtained from the Mwanza city in Tanzania. Lake Victoria is the major source of fish consumed by Mwanza residents and also receives treated wastewater effluents from Mwanza city. They previously described high rates of CTX-M15-producing *Enterobacteriaceae* in the city hospital as well as in animals and humans from the community (Mshana et al., 2016). In the present study, they reported a significant proportion of ESBL-producing *Enterobacteriaceae* in fish gut and environmental samples involving both clonal spread of resistant strains and dissemination of CTX-M15 encoding IncY plasmids. This study suggests that transmission of both ESBL-producing clones and plasmids may occur between humans and wild fish, and reciprocally, through environmental contamination by anthropogenic activity and the food chain, respectively. An additional article by Bai et al. highlights the diversity of ESBL-carrying plasmids, *bla*_CTX-M_ genes and ESBL-producing *E. coli* isolated from diarrheic patients in China. Thanks to whole genome sequencing, additional articles emphasize the important role and diversity of integrative elements implicated in multidrug resistance in animal and human pathogens, two articles from Bossé et al. and Bossé et al. and one from Simões et al., respectively.

Finally, two research articles investigated the resistomes of humans, cattle and fish in different environmental settings, by using a culture-independent approach on targeted resistance genes and/or associated mobile elements like integrons. Chainier et al. reported a high frequency of integron carriage in the gut of cattle and humans living in the same geographic area, in France and conversely Muziasari et al. described a low occurrence of resistance genes in the gut of farmed fish and in associated sediment samples in fish farms in the Northern Baltic Sea, in Finland. Using high throughput culture independent methods to study resistomes in different microbiota will provide useful information in the future to manage the spread of antimicrobial resistance.

In summary, this Research Topic addresses various issues related to the genetics of antimicrobial resistance in frame of the “One Health” concept. They emphasize the huge diversity of resistance genes, mobile genetic elements, and multidrug-resistant clones in different microbial environments. In the era of increasing antimicrobial resistance, judicious use of antibiotics is an absolute necessity in veterinary and human medicine to prolong antibiotic usefulness. On the other hand, bacteria have developed a large set of genetic weapons specialized in the acquisition and spread of acquired resistance genes. Successful MDR mobile genetic elements are one of the main driving forces in the antibiotic resistance burden. A research effort is needed to decipher the molecular basis of resistance dissemination within and between microbial niches to manage the evolution toward resistance.

## Author contributions

All authors listed have made a substantial, direct and intellectual contribution to the work, and approved it for publication.

## Conflict of interest statement

The authors declare that the research was conducted in the absence of any commercial or financial relationships that could be construed as a potential conflict of interest.
